# A Smartphone Camera-Based Indoor Positioning Algorithm of Crowded Scenarios with the Assistance of Deep CNN

**DOI:** 10.3390/s17040704

**Published:** 2017-03-28

**Authors:** Jichao Jiao, Fei Li, Zhongliang Deng, Wenjing Ma

**Affiliations:** 1 Beijing University of Posts and Telecommunications, Beijing 100088, China; li-fei@bupt.edu.cn (F.L.); dengzhl@bupt.edu.cn (Z.D.); 2 China Academy of Information and Communication Technology (CAICT), Beijing 100191, China; mawenjing@caict.ac.cn

**Keywords:** indoor positioning, smartphone camera, indoor crowded scenarios, population density, RSSI, deep CNN

## Abstract

Considering the installation cost and coverage, the received signal strength indicator (RSSI)-based indoor positioning system is widely used across the world. However, the indoor positioning performance, due to the interference of wireless signals that are caused by the complex indoor environment that includes a crowded population, cannot achieve the demands of indoor location-based services. In this paper, we focus on increasing the signal strength estimation accuracy considering the population density, which is different to the other RSSI-based indoor positioning methods. Therefore, we propose a new wireless signal compensation model considering the population density, distance, and frequency. First of all, the number of individuals in an indoor crowded scenario can be calculated by our convolutional neural network (CNN)-based human detection approach. Then, the relationship between the population density and the signal attenuation is described in our model. Finally, we use the trilateral positioning principle to realize the pedestrian location. According to the simulation and tests in the crowded scenarios, the proposed model increases the accuracy of the signal strength estimation by 1.53 times compared to that without considering the human body. Therefore, the localization accuracy is less than 1.37 m, which indicates that our algorithm can improve the indoor positioning performance and is superior to other RSSI models.

## 1. Introduction

Radio Frequency (RF) signal-based indoor positioning and navigation have attracted a lot of attention because of the low system installation cost and the acceptable positioning accuracy [1]. Therefore, RF signals are used in many buildings.

To support these location-based service (LBS) applications, many scholars have made great efforts to achieve accurate and robust indoor pedestrian positioning. Various indoor positioning technologies based on RF signals can provide an absolute position, such as Wireless Local Area Net (WLAN, also known as WiFi), cellular networks, Bluetooth, Radio Frequency identification (RFID) tags, and ZigBee, etc.

The general RF positioning approaches include fingerprinting [2,3] and triangulation [4]. The tedious database training is the main challenge for fingerprinting. The triangulation-based technology uses the geometry of triangles to obtain the relative positions of humans, so it requires three base stations (or more) with known coordinates. Moreover, a signal propagation model in indoor environments is needed to convert the received signal strength (RSS) to the distance between the access points (APs) and a user’s device (such as a smartphone). However, it is difficult to find an accurate model, because the transmitted signals suffer obstructions and reflections, which lead to wireless signal path losses [5]. All fingerprinting and triangulation technologies need to obtain exact RSS to prepare for the next positioning step. Moreover, the RSS value depends on the specific propagation environment. Especially in complex indoor environments, obstacles such as walls, floors, furniture, and people can contribute to the reflection, diffraction, scattering, and absorption of the signal. The existing path loss models have considered the influence factors, such as different types of walls and floors in buildings [5,6,7]. However, as we know, a high population density can greatly affect the transmission of the signal and decrease the positioning accuracy in crowded indoor settings, and this is not taken into account [8,9]. In some crowded environments, including bus stations, airports, and train transportations, the waveguides of wireless signals at 2.4 G or 5 GHz frequencies declined, because of the crowded passengers [10]. According to this research, the human bodies absorbed the wireless signals, especially the high frequency signals. Meanwhile, Gandhi and his colleagues stated that humans reflected the wireless signals at 5 G and 60 GHz frequencies, when the linear reflection coefficient was 0.4 [11]. Cotton and his co-authors implied that human body shadowing in off-body channels occurs when the body partially or completely occludes the direct signal path between a wearable wireless device and a nearby transceiver [12].

In this paper, considering the effect of people indoors and the rapid growth in the use of smartphones with a powerful integrated RGB-camera, we propose a new signal attenuation model to compensate for the propagation loss, based on 2-D information. In our model, the population density is regarded as an influencing factor, which is added to the traditional indoor signal propagation model.

The rest of the paper is organized as follows. In Section 2, we present several related works of signal attenuation estimation. In Section 3, we describe the proposed algorithm and an improvement by fusing image information. Both algorithms do not rely on the knowledge of distance-related information. In Section 4, we show the numerical simulations which are achieved in a real environment. Finally, conclusions and suggestions for further work are summarized in Section 5.

## 2. Related Work

Pioneering work by [13] quantitatively demonstrated that signal attenuation was affected by the human body. When the human body covers a smartphone, the signal is weakened and the RSS values drop by about 5–8 dB. Moreover, the increase in the number of individuals can also result in signal attenuation. Therefore, it is necessary to understand the relationship between the density of a population and signal attenuation. In [14], Kenichi Ito and Yu Hotta conducted experiments to analyze the signal path loss over several signal frequencies and signal transmission distances, respectively. Using the resulting curve, they clearly found a relationship between signal attenuation, and different frequencies and distances. Although some of the above studies have analyzed the impact of personnel on the signal path loss, those methods are only applicable to sparse scenarios, and cannot be applied to indoor positioning under crowded backgrounds.

In indoor locations, signal path loss has been well investigated in high-density crowds [15,16]. Hung-Huan Liu proposed a heuristic AP RSSI shaping algorithm to compensate for the signal attenuation caused by multipathing and shadowing [17]. Moreover, the attenuation factor of the main obstacles contains internal barriers. Similarly, an empirical propagation model, in which the neural networks were used to calibrate the Cheung model, to identify the relationship between signal power attenuation and observed phenomena like wall interactions and the transmitter-receiver distance, etc., has been developed by M. Ayadi and A. Ben Zineb [18]. Ahmadi and his co-authors proposed a training-less approach based on the real-time calibration of a simple path loss model for indoor positioning, based on mobile terminals [19]. Feng and his colleagues proposed an accurate RSS-based indoor positioning system using the theory of compressive sensing [20]. However, the above methods ignored the human activations.

By fusing the information obtained from the wireless receiving module and the smartphone camera with the novel signal propagation model, the indoor positioning accuracy based on the wireless network is improved for crowded scenarios.

## 3. Turbo RSSI Model-Based Indoor Algorithm for Crowded Scenarios

Based on the above discussion, we propose a new positioning method for high-density crowds. This method combines the location information from the wireless receiving module and the camera module which are integrated into smartphones. The signal path loss is compensated for with a high accuracy, by using the proposed signal propagation model. Moreover, CNN is used for calculating the population density by images captured by the smartphone camera. Experimental results show that the signal path loss was precisely compensated for, which achieved a sufficient indoor positioning accuracy in crowded scenarios.

### 3.1. RSSI Log-Distance Path Loss Model

For RSS-based positioning systems, the use of a proper radio propagation model is very important. In order to calculate the distance between two radio devices using RSS, we must select the appropriate radio propagation model. In outdoor spaces which include only a few obstacles, the free space propagation loss is the main path loss when radios transmit signals. However, in complex indoor situations, there are many obstacles including walls, furniture, and human bodies, between a transmitter and a receiver, resulting in signal loss. Equation (1) is commonly used for estimating the path loss [21].

Consequently, we can translate the unit in dB into dBm, which is used in RSSI, and we can estimate ξ to ascertain the distance by using Equation (1):

(1) $$P_{r}=P_{0}-10\alpha \mathrm{lg}d+\xi$$

where:

- $P_{0}$ is the RSSI of the transmitter.
- d is the distance between the receiver and the transmitter in the meter.
- α is known as the decay rate of the received signal level. In the free space, α=2. However, for obstructed paths located indoors, α is related to the specific environment.
- ξ is a Gaussian random variation whose mean is μ=0 in dB. The standard deviation σ in dB is due to shadow fading, which is determined by the actual measurement results.
- $P_{r}$ is the signal strength of the receiver.

Most statisticians would not view the least-squares estimation (LSE) as a general method for parameter estimation, but rather as an approach that is primarily used with linear regression models [22]. LSE can be treated as a special application of the maximum likelihood estimation (MLE) when the distribution is Gaussian [23]. In other words, when the distribution of the Path Loss Model parameters is Gaussian, we can use LSE to replace the MLE for estimating the parameters. However, in order to make our algorithm more robust, we introduce the MLE.

**Figure 1:**
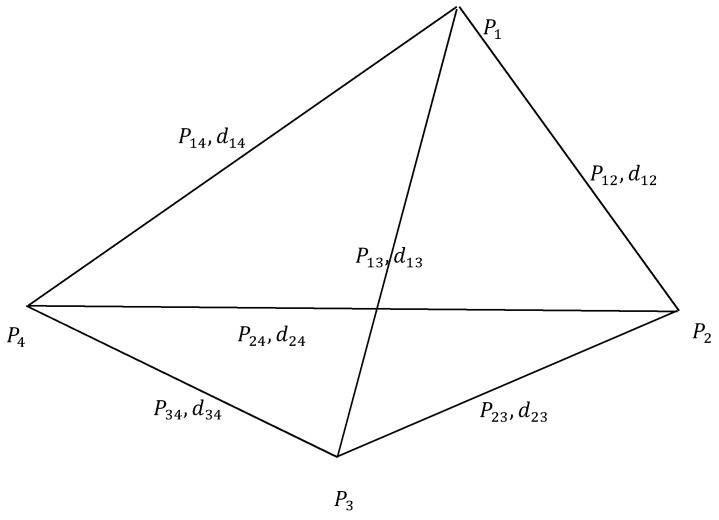
A fully-connected planar quadrilateral in sensor networks.

In order to estimate the path loss exponent that affects the indoor positioning accuracy of the fingerprinting-based wireless localization, α should be robust enough for the indoor environment. Therefore, inspired by [24] and according to Figure 1, the path loss exponent estimation problem can be regarded as the estimation of the true distances $d_{12},d_{13},d_{14},d_{23},d_{24},d_{34}$ between the user and Aps, when the power measurements $P_{12},P_{13},P_{14},P_{23},P_{24},P_{34}$ are given. $P_{0}$ is the RSS at distance $d_{0}$. If $P_{ij}\neq 0$, sensors i and j are neighbors. Moreover, the likelihood function can be gained by using MLE [25]:

(2) $$\begin{array}{l} L(d_{12},d_{13},d_{14},d_{23},d_{24},d_{34},\alpha )\\ =\frac{1}{{\left(\sqrt{2\pi }\sigma \right)}^{6}}\prod_{1\leq i<j\leq 4}\exp(-\frac{{\left(P_{ij}-P_{0}+10\alpha \mathrm{lg}d_{ij}\right)}^{2}}{\sigma^{2}}) \end{array}$$

For computational convenience, the MLE estimation is obtained by maximizing the log-likelihood function that is shown by Equation (2). However, it is a computational processing of maximizing Equation (2). Therefore, we minimize Equation (3), which is the log-likelihood of Equation (2). Maximizing L is equivalent to minimizing $g(d_{12},d_{13},d_{14},d_{23},d_{24},d_{34},\alpha )$, where:

(3) $$\begin{array}{l} g(d_{12},d_{13},d_{14},d_{23},d_{24},d_{34},\alpha )\\ =\sum_{1\leq i<j\leq 4}{\left(P_{ij}-P_{0}+10\alpha \mathrm{lg}d_{ij}\right)}^{2} \end{array}$$

In practice, however, it is usually not possible to obtain an analytical form solution for the MLE estimate, especially when the model involves many parameters and its probability density function is highly non-linear. Therefore, in our proposed algorithm, the Cayley–Menger determinant is used to constrain the space of searching for the MLE. When considering positioning, the Cayley–Menger determinant is always used in distance geometry for determining the volume of a triangular pyramid based on the distances between any two of the four vertices [26,27]. Therefore, based on the Cayley–Menger determinant, the local minimization is achieved. The determinant of a quadrilateral is shown by the following function:

(4) $$D(P_{1},P_{2},P_{3},P_{4})=\left|\begin{array}{ccccc} 0 & d_{12}^{2} & d_{13}^{2} & d_{14}^{2} & 1\\ d_{12}^{2} & 0 & d_{23}^{2} & d_{24}^{2} & 1\\ d_{13}^{2} & d_{23}^{2} & 0 & d_{34}^{2} & 1\\ d_{14}^{2} & d_{24}^{2} & d_{34}^{2} & 0 & 1\\ 1 & 1 & 1 & 1 & 0 \end{array}\right|=0$$

When $D_{ij}=P_{{}_{ij}}/P_{0}(1\leq i<j\leq 4)$ is known, a nonlinear equation of α can be obtained by combining Equations (3) and (4), which is shown as follows:

(5) $$p(\alpha )=\left|\begin{array}{ccccc} 0 & D_{12}^{-2/\alpha } & D_{13}^{-2/\alpha } & D_{14}^{-2/\alpha } & 1\\ D_{12}^{-2/\alpha } & 0 & D_{23}^{-2/\alpha } & D_{24}^{-2/\alpha } & 1\\ D_{13}^{-2/\alpha } & D_{23}^{-2/\alpha } & 0 & D_{34}^{-2/\alpha } & 1\\ D_{14}^{-2/\alpha } & D_{24}^{-2/\alpha } & D_{34}^{-2/\alpha } & 0 & 1\\ 1 & 1 & 1 & 1 & 0 \end{array}\right|=0$$

Following this, α and the path loss model are determined. This model calculates the path loss based on the propagation attenuation in the free space that is accumulated by the attenuation due to obstacles. However, a disadvantage of this model is that the presence of people is not taken into account. We will consider effects such as people shadowing in our new signal attenuation model.

### 3.2. The Effect of the Human Body

For indoor scenarios, many short-range wireless communication devices rely on a direct path for wireless transmission. However, the direct path is easily blocked by human bodies, so we should fully consider the shielding factor of the human body on the electromagnetic wave propagation.

All kinds of human organs are lossy mediums, so the human body will induce an electromagnetic field and generate a current under the external electromagnetic field. Therefore, it can absorb and dissipate electromagnetic energy [28]. The Specific Absorption Rate (SAR) is commonly used in the biological metrology to characterize the physical process. SAR (*W*/kg) is the electromagnetic power that is absorbed or consumed by the bodily tissue [29].

We leverage the Finite-Difference Time-Domain (FDTD) [30] to analyze the electromagnetic distribution in the human body. Electromagnetic distribution analyzation is a necessary procedure for estimating the affection of the human body on the wireless positioning signals. The finite element method (FEM), a method of moments (MoM), and FDTD are widely used in the electromagnetic distribution analyzation. Thanks to Davidson’s work [31], we know that FDTD is the only technique where one person can realistically implement oneself in a reasonable time frame, but even then, this results in quite a specific problem, as proposed in this paper. The other four field components can be obtained in the same way, by functions that were proposed in [32].

It is noted that the electromagnetic field includes two aspects: electric and magnetic fields. The living organisms of human bodies can affect the range of those two fields. The human body can be assumed as a conductor. In this context, when the wireless signal passes through the human body, charges gather on the skin as it remains in the electric field, and the spatial distribution of the original electric field of the human body is changed [33]. Therefore, the power of the wireless signal attenuates.

Considering that the electrical conductivity and the dielectric constant are different in various tissues and organs, the average SAR is calculated by the following formula:

(6) $$SAR_{AV}=\frac{1}{2}\frac{\int \sigma (v){\left|E(v)\right|}^{2}dv}{\int \rho (v)dv}$$

where:

σ is the conductivity of each grid cell for the tissue and is in units of (S/m); S is the capital letter of Siemens that is a standard International Unit of electrical conductance.

E(v) represents the electric field intensity value for biological tissue.

ρ is the biological tissue mass density and is in units of kg/m^3^.

$E(v)=\sqrt{E_{x}^{2}+E_{y}^{2}+E_{z}^{2}}$, and the units of (V/m) and V (voltage), are the derived units for electric potential, electric potential difference, and electromotive force, respectively.

### 3.3. Human Detection

In order to detect the human body, we introduced the deep CNN [34] to local humans and calculated the number of humans. Human detection methods can be classified into two categories, based on human feature descriptors [35]. One category is based on the pre-designing descriptors, that include scale invariant feature transformation (SIFT) [36], histograms of oriented gradients (HOG) [37], local binary patterns (LBP) [38], and so on. Before detecting the human, the user must choose an appropriate feature based on their previous knowledge. Generally, each pre-designing feature can achieve a good performance in one or several application backgrounds. For example, SIFT-based methods achieve a remarkable performance in the scalable application with computational work [39]. HOG is widely used in occlusion detection without much illumination, and LBP can work well in scenarios of real-time detection without occlusion [40]. However, some challenges, such as the occlusion, illusion change, and real-time scenarios, widely and simultaneously exist in indoor environments. Moreover, it is necessary to use a classifier in the human detection methods based on pre-design features [41], which makes the detection methods more complex. Therefore, in order to make our human detection robust, we introduce a CNN-based method that can create an image descriptor, according to the scene.

**Figure 2:**
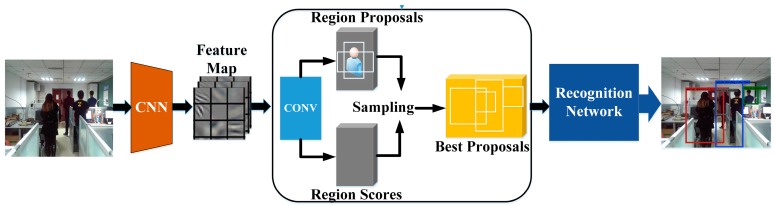
Flowchart of human detection.

In order to detect the human body, we introduced the deep CNN [42] to local humans and calculated the number of humans. The CNN-based method generalizes object detection, where a signal word is used to describe the region and image captioning, and the image is covered by a full region, with a short sentence describing it. This technique is based on a fully convoluted localization neural network (FCLN) architecture that includes a convolutional neural network, a dense localization model, and a recursive neural network language model, which is shown by Figure 2. However, in order to speed up this processing, the recursive neural network language model was removed from our algorithm. The architecture of the convolutional neural network is formed by the following:

(7) $$\begin{array}{l} s(i,j)=(X*W)(i,j)+b\\ \begin{array}{c} \;=\sum_{k=1}^{N_{matrix}}(X_{k}*W_{k})(i,j)+b \end{array} \end{array}$$

where $N_{matrix}$ is the number of input matrices, $X_{k}$ is the $k^{th}$ input matrix, $W_{k}$ is the $k^{th}$ convolutional kernel, b is the offset, s(i,j) is the output value at position (i,j), and output matrix s is the feature map.

Convolutional processing forms the basis of the CNN and performs the core operations of training, and consequently, the firing of neurons in the network. After off-line training, FCLN is robust in detecting humans, which is improved through various simulation results in Section 4.

The convolutional network adopts visual geometry group-16(VGG-16) architecture to produce the feature maps of original images. The following localization layer receives these activations, identifies spatial salient regions, and separates a scale-invariant representation from each region. The recognition network is a fully-connected neural network which processes the region feature received from the localization layer. For each region, this produces a code of dimension $Dim=w_{con}\times h_{con}\times v_{feature}$ flattened from the features, in which $w_{con}$ is the width of the convolutional kernel and $h_{con}$ is the height of the convolutional kernel. $v_{feature}$ is the dimension of the image features.

The flowchart of human detection is shown in Figure 2. The original image is first processed by a convolution network. Then, the localization layer generates region proposals and smoothly extracts a batch of corresponding activations using bilinear interpolation. The activation of region features are finally sent to the recognition network, to identify the people in the image. Finally, the computer tells us how many people are situated on the signal propagation path.

It is noted that the key layer of FCLN is the human localization layer, which is marked in Figure 2 [42]. From Figure 2, we can see that the human localization layer proposes regions and smoothly extracts a batch of corresponding activations using cubic interpolation. These regions are processed with a fully-connected recognition network and described with a recurrent neural network model [35]. The model is trained end-to-end with gradient descent [42]. Moreover, we can ascertain that the region proposals of the human localization layer are used to produce an initializing region for the human region candidates. The initialized size of each region R is $\frac{W_{img}}{w_{con}}\times \frac{H_{img}}{h_{con}}$ ($W_{img}$ is the width of an image, $H_{img}$ is height of an image), and the coordinates of each region, are described as follows:

(8) $$x=x_{cen}+l_{x}W_{R}$$

(9) $$y=y_{cen}+l_{y}H_{R}$$

(10) $$w=W_{R}\exp(t_{w})$$

(11) $$h=H_{R}\exp(h_{w})$$

where (x,y) is the center of the output region, $R(x_{cen},y_{cen})$ is the center of the initialized region, $\left(l_{x},l_{y},l_{w},l_{h}\right)$ is the predicted scalars of the FCLN, and $W_{R}$ and $H_{R}$ are the width and height, respectively.

(12) $$\begin{array}{l} W_{R}=\frac{W_{img}}{w_{con}}\times \frac{1}{2}\\ H_{R}=\frac{H_{img}}{h_{con}}\times \frac{1}{2} \end{array}$$

Based on calculating the region scores, which are the vectors of length w, a confidence score for each output region is produced, and this is used for locating each human.

After training the FCLN by using our database, we can build a feature-map model for detecting and recognizing humans. Then, the humans in a picture taken by a user can be located when the picture is sent to the server installed in the FCLN. Then, each person in the image can be located by the regions $B^{\prime }\left\{b_{1}^{\prime },b_{2}^{\prime },b_{3}^{\prime }....,b_{n}^{\prime }\right\}$ of the human localization layer. Finally, we can obtain the human number n by calculating the number of output regions.

### 3.4. New Indoor Propagation Model Considering Human Body

We use the human body penetration loss model to modify the Log-Distance Path Loss Model, and the indoor radio signal propagation model is then obtained. The model compensates for the power loss caused by people and obstacles for the received attenuation signal, and we can then obtain the transmission power. The new model is formulated as follows:

(13) $$RSS=RSS_{re}+PL_{human}+PL(d)$$

where:

RSS: the transmission power.
$RSS_{re}$: the strength of the actual received signal in (dBm).

The transformation formula of (dBm) and (W) is presented in the following function, and *W* is a derived unit of power in the International System of Units:

(14) $$\begin{array}{l} PL_{human}=10\mathrm{lg}\frac{P\times 10^{3}(mw)}{1(mw)}\\ \;=30+10\mathrm{lg}(human_{weight}\times n\times SAR_{AV}) \end{array}$$

where $PL_{human}$ is the dissipation of the signal power caused by human bodies in (dBm), while P is the dissipation of the signal power caused by human bodies in (*W*). $human_{weight}$ is the average weight of a human in a unit of kg. n represents the number of people in the occlusion, and this number can be detected from the picture taken using the smartphone camera.

The new model is named the Turbo RSSI model, which is used to calculate the distance from the transmitter node to the receiver node for indoor positioning.

### 3.5. RSS-Based Positioning

In this paper, a three-border positioning method is introduced to estimate the distances from three or more transmitters to the final position of the device [4].

**Figure 3:**
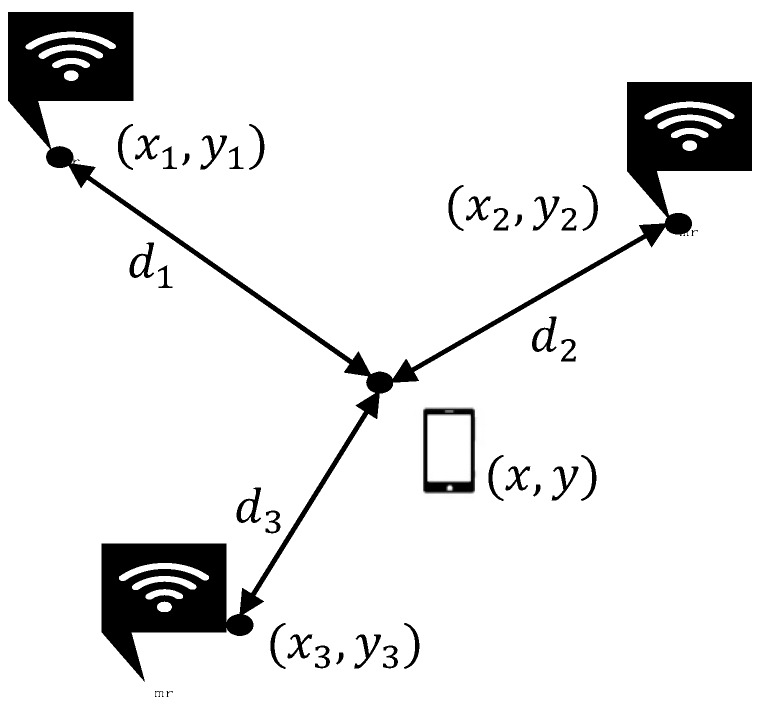
Three-border positioning.

As shown in Figure 3, we can easily ascertain the coordinates of the reference nodes $A(x_{1},y_{1})$, $B(x_{2},y_{2})$, and $C(x_{3},y_{3})$, and the distances $d_{1},d_{2},d_{3}$. If we assume that the coordinates of the user with a smartphone are (x,y), the Equation (15) can be established:

(15) $$\{\begin{array}{c} {\left(x-x_{1}\right)}^{2}+{\left(y-y_{1}\right)}^{2}=d_{1}{}^{2}\\ {\left(x-x_{2}\right)}^{2}+{\left(y-y_{2}\right)}^{2}=d_{2}{}^{2}\\ {\left(x-x_{3}\right)}^{2}+{\left(y-y_{3}\right)}^{2}=d_{3}{}^{2} \end{array}$$

The indoor location of the smartphone can be obtained by Equation (15).

### 3.6. Steps of Our Indoor Positioning Algorithm

**Figure 4:**
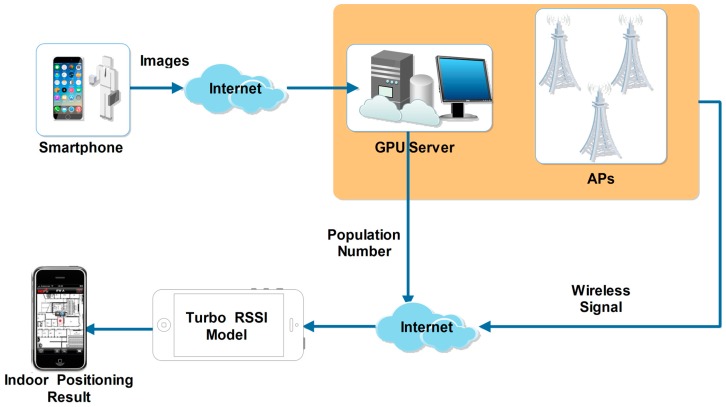
Flowchart of our proposed indoor positioning algorithm based on the Turbo RSSI Model.

According to the above analysis, the implementation of our proposed algorithm is shown in Figure 4. Being indicative of the flowchart, the steps of our algorithm are described by the following procedure:

(1) The user takes an indoor image I(x,y) which includes pedestrians, using the camera of a smartphone;
(2) I(x,y) is sent to our GPU server, and the individual number n of the I(x,y) is calculated based on FCLN, by using Equation (7). It is worth noting that in our experiment $w_{con}=32$, $h_{con}=32$;
(3) n is sent back to the smartphone;
(4) The WiFi signals $P(i)=\left\{p_{1},p_{2},...,p_{i}\right\}(i\geq 3)$ from APs are received by the smartphone;
(5) n and P(i) are introduced in the Equation (14) that runs on the smartphone, which is used to compensate for the signal strength loss $PL_{human}$. It is noted that the human weight $human_{weight}=60$.
(6) $PL_{human}$ is used in the Equation (3), and then the distances $D=\{d_{1},d_{2},...,d_{i}(i\geq 3)\}$ are estimated.
(7) *D* is introduced in the Equation (15), and then the indoor location (x,y) of a user who takes a smartphone photograph is calculated, for which the accuracy is less than 2 m.

## 4. Tests and Evaluation

### 4.1. Experimental Setting

**Figure 5:**
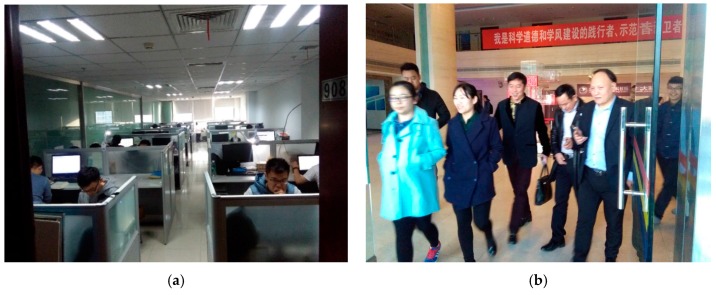
Indoor scenarios of our experiment. (**a**) Room 908; (**b**) Hall of our research building.

**Figure 6:**
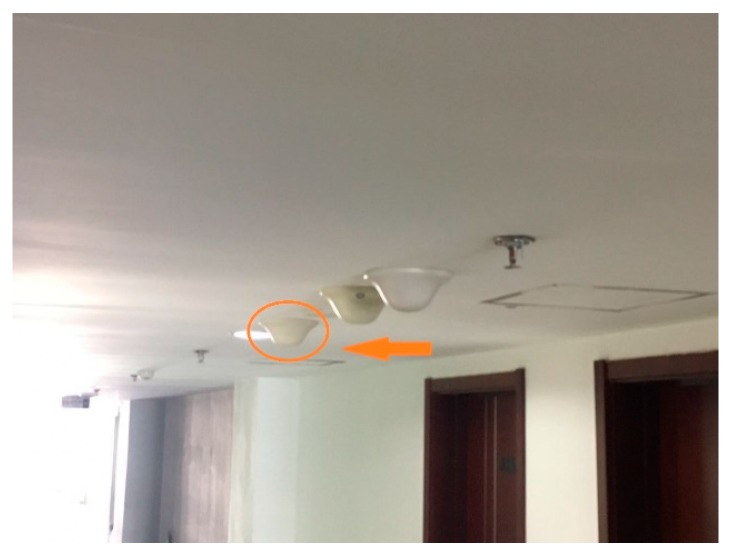
A signal transmitter in the corridor.

**Figure 7:**
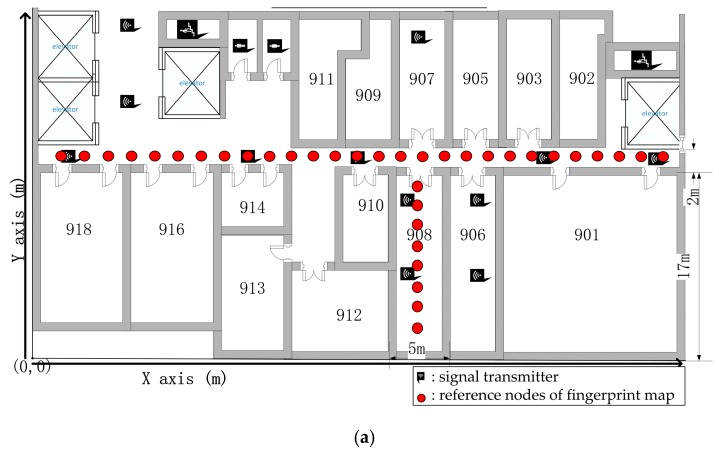

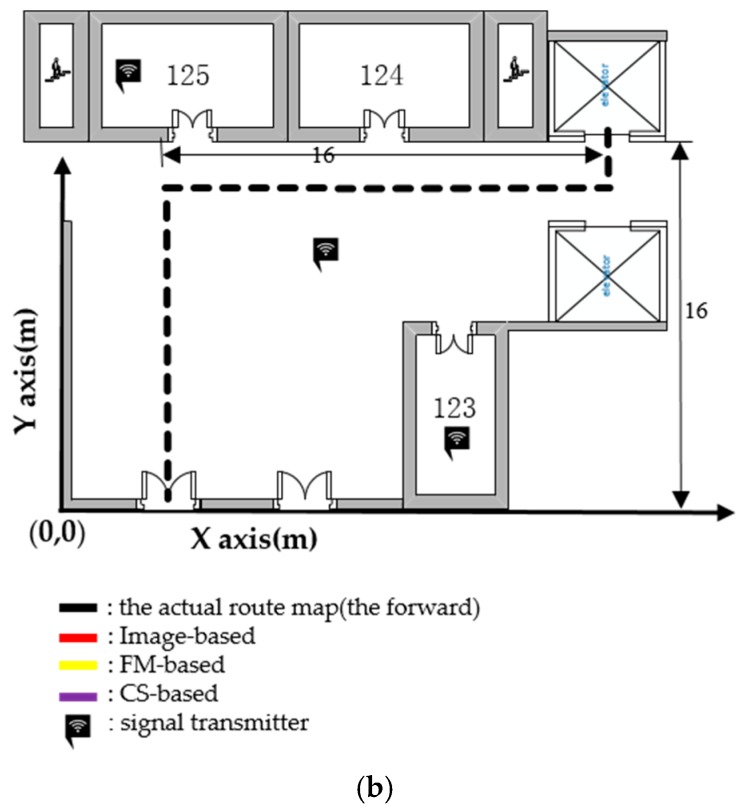
Test path in Room 908 and the hall. (**a**) Room 908; (**b**) Hall of our research building.

The experiments are achieved at the New Research Building of Beijing University of Posts and Telecommunications (BUPT). The 9th floor of the building has a main corridor which is 2 m wide and 60 m long, and room 908 is about 5 × 17 m^2^. These two areas are mainly used for testing our proposed algorithm. This test environment is shown in Figure 5. Moreover, in the test environment, we assume that people are evenly distributed, but it is a crowded space, where there are two individuals in 1 m^2^, as shown by Figure 6. It is noted that there are four people in 1 m^2^ when we test our proposed algorithm. Moreover, we produced the pre-design paths in Figure 7 for comparing them to the results based on the other three indoor positioning methods.

**Figure 8:**
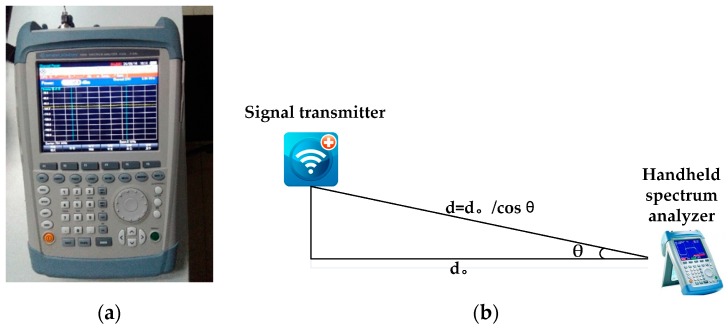
(**a**) Measurement equipment: handheld spectrum analyzer and (**b**) an example for experiment setup.

Our research building is a large building with well-equipped infrastructure. There are indoor omnidirectional ceiling antennas that are called the signal transmitters, which form a supplementary system of the base station signal inside the building. The signal transmitter can send the indoor positioning signals. Figure 6 shows the signal transmitter, which is mounted 2 m above the floor. It exhibits a 754 MHz frequency band. The X and Y coordinates of the 12 APs shown in Figure 8 are set up around the 9th floor, which are AP1(4,18), AP2(13,23), AP3(13,30), AP4(20,18), AP5(30,18), AP6(35,7), AP7(35,15), AP8(37,28), AP9(42,7), AP10(42,15), AP11(48,18), and AP12(58,18), and the coordinate origin (0,0) is in the lower left corner of Figure 8. We detect the signal power in d = 0 is −20 dBm.

In order to evaluate the proposed method, an Agilent CSA N1996A (Keysight Technologies Inc., Santa Rosa, CA, USA) acting as an RF signal generator has been used, and the Agilent FieldFox N9912A portable spectrum analyzer (Keysight Technologies Inc., Santa Rosa, CA, USA) is used as a receiver, as shown in Figure 8.

### 4.2. Human Detection Evaluation

According to our research, and considering the realistic indoor environments, we have given the human number calculation results in three different scenarios that include a small office room, building lobby, and corridor. Moreover, we took images in the morning and evening for each scenario, which was used to improve the illumination invariance of our method. Therefore, 300 images were taken for each scenario. It is noted that we introduced the counting error to evaluate the performance of our method, which is expressed by the following formula:

(16) $$\kappa =\frac{\left|N_{FCLN}-N_{manul}\right|}{N_{manual}}$$

where $N_{FCLN}$ is the number of humans identified by using the FCLN, and $N_{manul}$ is the number of humans counted by a human. The experimental results are shown as follows.

**Figure 9:**
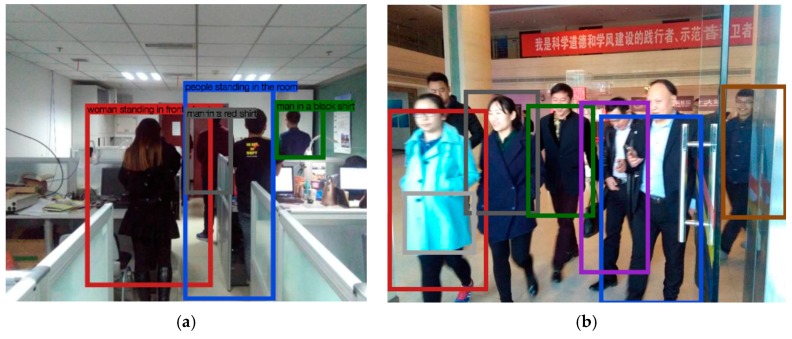
Human detection. (**a**) Room 908; (**b**) Hall of our research building.

**Figure 10:**
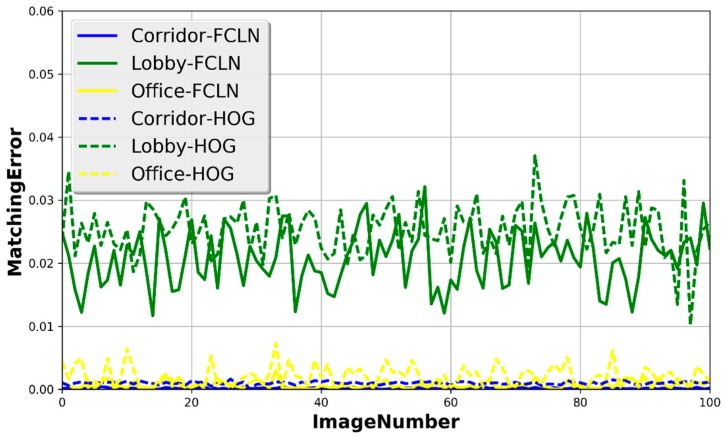
Matching errors in three indoor scenarios.

We compared the results from the following techniques or combinations: (1) our proposed FCLN; (2) HOG that is widely used in human detection; (3) the ground-truth data that are manually calculated. Figure 9 and Figure 10 demonstrate the counting error rate in different scenarios. According to Figure 10, we can ascertain that the κ in the corridor is the best among the three indoor scenarios, because the shape of the corridor makes it easy to count the human number. κ in the lobby is the worst among the three indoor scenarios because of its wide space where many people walk, which makes it difficult to detect the number of people. Additionally, our proposed FCLN can achieve a better result than HOG when the number of humans, which is useful for estimating the path loss with a high accuracy.

### 4.3. Turbo RSSI Model Evaluation

**Figure 11:**
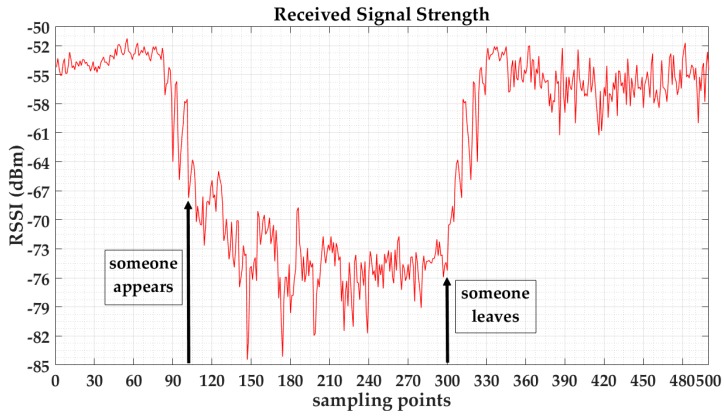
Relationship of the human and the received wireless signal strength.

We first test whether the emergence of personnel will affect the signal transmission. In this section, the human body is tested for the influence of RSSI readings. We took the handheld spectrum analyzer at position (24,18). Then, we collected the RSSI of AP4 with 100 sampling points; there were then several people between AP4 and our measuring instrument, and we collected the RSSI with 200 sampling points at the same position; following this, people left, and a further 200 sampling points were collected. Figure 11 shows the variation of the received signal strength, according to the human walking. According to Figure 11, we can see that the RSSI curve is relatively stable when no person appears, but when someone appears, the curve appears to fluctuate significantly. The signals are weakened, which makes the curve of the received signal strength dramatically change, and this is marked by arrows.

**Figure 12:**
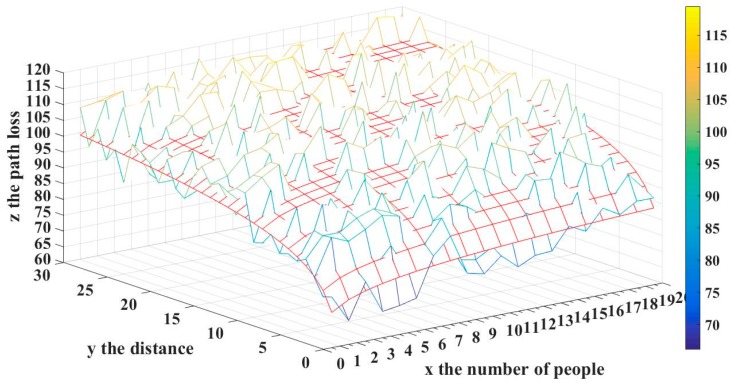
Signal attenuation with respect to distance and the number of people.

We use path loss model Equation (14) to generate the RSSI of each AP. Moreover, there is noise from the wall or furniture, so we add Gaussian noise N(0,5) in ξ of the path loss model for simulating the noise. The fingerprint map in the building with 36 reference nodes and a distance of 2 m for each point is shown in Figure 7. In our experiment, a different number of people are standing uniformly between the transmitter and the receiver. We obtain the RSS values for the different distances. According to our model, the attenuation results of a different number of people and the different distances between the transmitter and the handheld spectrum analyzer are shown in Figure 12.

Figure 12, with x as the number of people who block the signal transmission and y as the distance between the signal transmitter node and the receiver node, implied the relationship between the signal attenuation and attenuation factors such as the distance from the transmitter node to the receiver node and the number of people according to our model. Besides, the red grid line represents the theoretical value; while the other represents the actual measured value. The attenuation model for the presence of a different number of people is derived, based on the logarithmic curve shown in Figure 12, which suggests that the theoretical value of our model approximates the experimental measurement.

**Figure 13:**
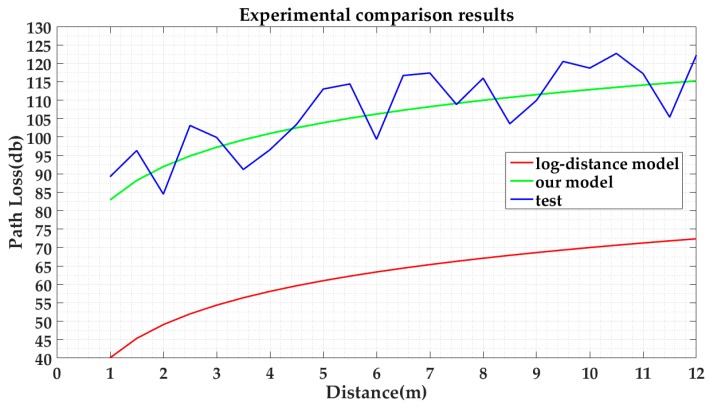
Comparison of our model, the Log-Distance path loss model, and the ground-truth data.

Figure 13 illustrates a comparison between our proposed model, the Log-Distance path loss model, and the practical measured values. It is worth noting that all points are measured on the condition that the number of people is two. According to Figure 13, we can learn that the curve of our model is more approximate to the measured values than that of the Log-Distance path loss model without considering humans.

**Figure 14:**
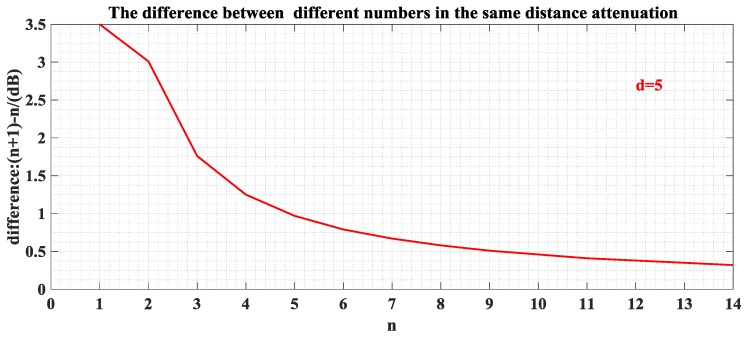
Relationship of the signal strength and the human number at a fixed distance (d = 5 m).

In the experiment, we find that when the number of people reaches a certain level, the change in the signal attenuation caused by personnel factors is no longer obvious. Figure 14 shows the difference in signal attenuation for each additional person at a distance of five meters (the initial number is one person). As the number increases, the difference in signal attenuation gradually decreases. Therefore, the influence of the number of humans will no longer change significantly when it is bigger than 13, and the difference will be infinitely close to zero.

### 4.4. Positioning Performance

In order to evaluate the performance of our proposed method, two state-of-the-art indoor positioning methods were introduced. The first method is based on our proposed method that estimates the RSSI, considering humans. The second method is used to estimate the CrowdSourcing RSSI of the WiFi network (CS-based or Zee) [43]. The final method is employed to calculate the RSSI of the FM broadcast signal, without considering human affection (FM-based) [44]. In our experiment, a smartphone and a handheld spectrum analyzer are used to evaluate the accuracy and robustness of the positioning methods used in this paper. A 5 dB gain monopole antenna has been connected to both the transmitter and the receiver. The initial parameters of our smartphone are shown in Table 1. With many people walking around randomly, we walked straight along the corridor, then entered room 908, and went straight along the corridor. We took pictures to detect the number of people and to test the RSS values from several meters away. The positioning results are shown in Figure 14.

**Figure 15:**
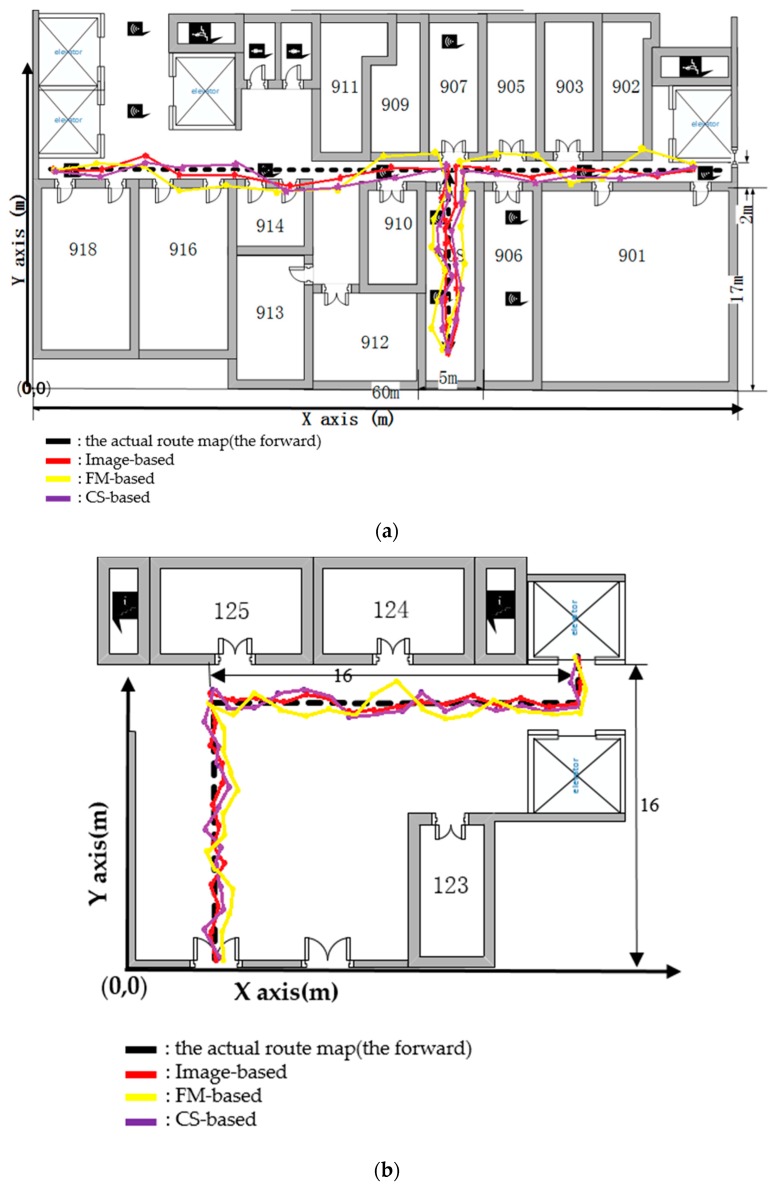
Position results when using different strategies in different indoor scenarios. (**a**) Room 908; (**b**) Hall of our research building.

Figure 15 shows that both the forward and backward results had a similar shape when using the different positioning methods in the three indoor scenarios, but suffered from long-term drifts. The drifts were triggered by signal attenuation, such as human body absorption, multipathing, and other factors. The red curve shows that the user’s locations in the corridor are precisely estimated. However, some erroneous positioning points are calculated based on the RSSI Log-Distance Path Loss Model method and the fingerprint method, which are shown by the yellow curve and purple curve in Figure 15, respectively.

In order to characterize the positioning accuracy, the truth positions were taken in advance, and the estimated locations were then compared to them. The root mean square error (RMSE) is introduced to evaluate the performance of the proposed algorithm. The positioning accuracy in RMSE can be computed between the real ground point and its estimated positioning result. The comparisons of the estimation accuracy are listed in Table 2.

**Figure 16:**
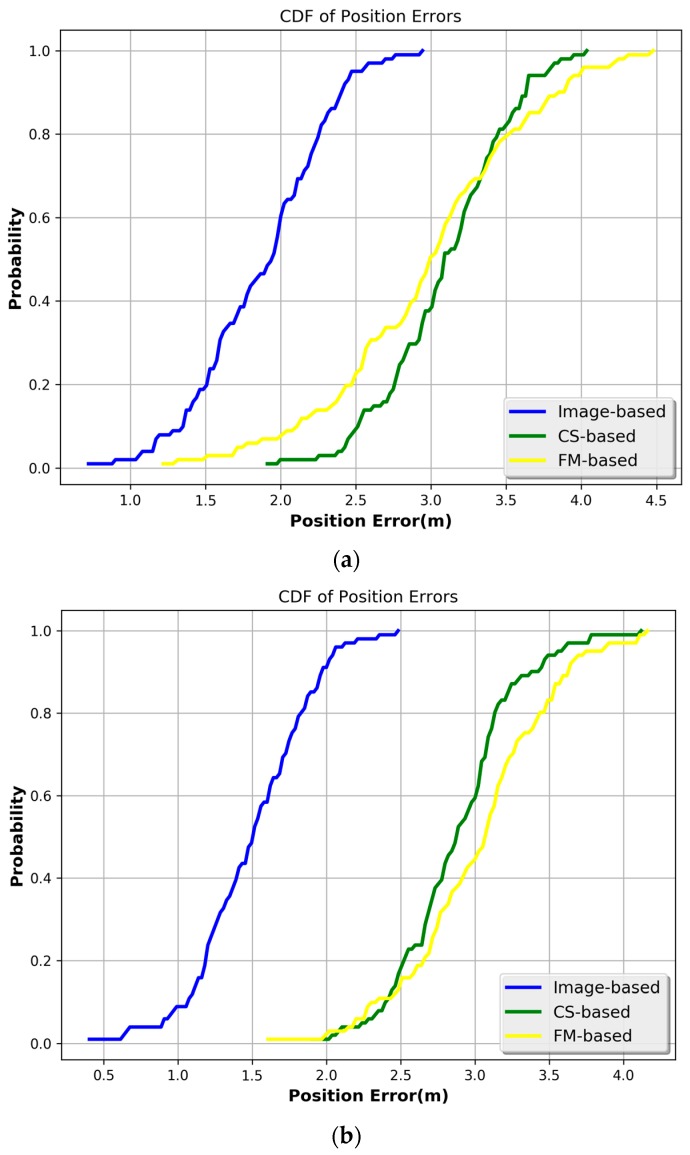

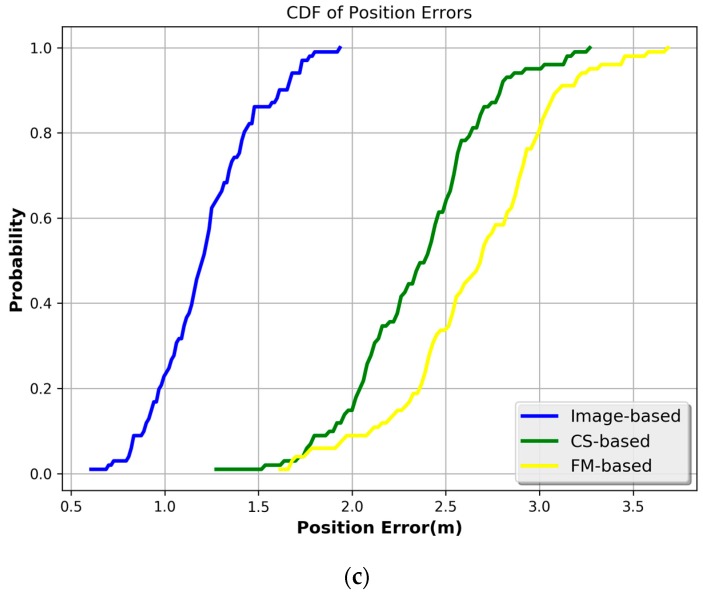
CDF of position errors when using different strategies. (**a**) Lobby; (**b**) Room 908; (**c**) Corridor.

To summarize, Figure 16 provides the cumulative distribution function (CDF) curves of the position errors.

Figure 16 indicates that the positioning results in the corridor are better than those in the office and lobby, which is consistent with the path loss reuslts. In the lobby, the positioning error is under 2.25 m (1σ) based on our proposed model. Besides, the positioning error obtained by the FM-based method is under 3.20 m (1σ), and the positioning error obtained by the CS-based method is under 3.30 m (1σ). Moreover, in the office, the positioning error is under 1.75 m (1σ) based on our proposed model., while it is under 3.02 m when obtained by the CS-based method, and is under 3.09 m (1σ) when obtained by the FM-based method. Furthermore, in the corridor, the positioning error is under 1.35 m (1σ) based on the proposed model, while it is under 2.72 m (1σ) based on the CS-based, and is under 2.87 m (1σ) based on the FM-based method. Therefore, we can see that our proposed model can achieve a better performance in terms of the positioning error than the other two methods, which implies that our model is robust for the different indoor scenarios.

In Table 2, we illustrated the whole performance of the three methods by demonstrating the statistical values of the errors (1σ) in the three scenarios. From the comparison results, we can show that the proposed algorithm is outperforms the CS-based and FM-based methods. Our positioning algorithm is highly robust and can achieve an accurate estimation, with an RMSE of 1.14 m. The comparison result indicates that the model considering the presence of people could obtain a better accuracy than that based on the other methods in dense crowds.

## 5. Conclusions

In this paper, we have presented an approach to model signal attenuation during RSSI, as a means of producing results with a high accuracy for indoor localization in crowded scenarios. Since there is a significant impact on the signal fluctuations when the number of individuals is taken into consideration, we added the shadowing factor of human presence to the signal attenuation model and combined the image-based method to obtain the factor ***n***—the number of characters. Based on the data measured by the smartphone, our Turbo RSSI model can predict the precise distance between the wireless signal transmitter node and the receiver node. Then, we leveraged the three-border positioning method to obtain the user’s position. In addition, the RSSI radio demonstrates a possibility for improvement, with a compensation based on the presence of people. By comparing it to the other two methods, the results showed that we improved the positioning accuracy in the crowded environments.

It is noted that we completed the tests in an experimental condition. In the future, we will improve our algorithm so that it can be used in different kinds of indoor scenarios that include the shopping mall, the public transportation center, and so on. Moreover, our proposed algorithm only asks each user to take an indoor picture when he/her needs the navigation service in a new indoor scenario. Besides, the locations of the transmitting stations are collected, before supporting the navigation and positioning services to users. Moreover, we are planning to develop a simple but powerful human detection approach which can run on the smartphone, not the server. Therefore, the indoor positioning method based on the Turbo RSSI model would be able to be run on the smartphone, without the need for servers. Therefore, we leave these extensions to future work.

## Figures and Tables

**Table 1:** The key technical parameters.

Parameter	Value
Camera	13 MP
Sampling period	2.0 s
The initial RSSI value	−20 dBm
Image resolution	2048 × 2048 pixels
The transmitter height	2 m

**Table 2:** Performance comparison of the accuracy (1σ).

Algorithm	Min Error (m)	Max Error (m)	RMSE (m)
Image-based	0.52	1.37	1.14
CS-based	1.23	3.98	2.37
FM-based	1.06	2.85	2.06
